# Experiences and Perceptions of Patient Watch, a Rural Telehealth Case Management Model, for Frequent Presenters to the Emergency Department: A Longitudinal Mixed Methods Study

**DOI:** 10.1111/ajr.70232

**Published:** 2026-07-11

**Authors:** Mary Malakellis, Anna Wong Shee, Laura Alston, Vincent L. Versace, Pheona Griffith, Jade Odgers, Kevin Mc Namara

**Affiliations:** ^1^ Deakin Rural Health Deakin University Warrnambool Victoria Australia; ^2^ Grampians Health Ballarat Victoria Australia; ^3^ Research Unit, Colac Area Health Colac Victoria Australia

**Keywords:** case management, emergency department, frequent presenters, mixed methods, rural, telehealth

## Abstract

**Objective:**

To evaluate: (1) patient experiences and perceptions of Patient Watch; and (2) the effect on emergency department (ED) presentations, healthcare use, quality of life and chronic illness care.

**Design:**

A prospective longitudinal cohort study using a convergent‐parallel mixed methods study design combining: (1) qualitative semi‐structured interviews analysed using reflexive thematic analysis and (2) quantitative assessment of Quality of Life‐8D, Patient Assessment of Chronic Illness, routinely collected ED clinical data and Patient Watch programme data.

**Setting:**

A large public health service delivering care across the home, community, aged care and hospital, serving a regional centre and small, medium and large rural towns in Western Victoria, Australia.

**Participants:**

Forty‐five participants were enrolled in the study. A subsample who completed all study requirements enabled baseline and 6‐month follow‐up analysis (*n* = 37).

**Intervention:**

Patient Watch, a telehealth case management model of care adapted from a metropolitan to a rural context, to manage the care of frequent presenters to the emergency department.

**Results:**

Before enrolment, patients reported health system, medical and situational complexity hindered effective care and contributed to high treatment burden, with acute exacerbations leading to increased healthcare use and negative care experiences. Patient Watch improved perceived care coordination, healthcare access and helped participants manage acute exacerbations. ED presentations (*p* < 0.001), hospital admissions (*p* < 0.001), general practitioner (*p* = 0.02) and specialist visits (*p* = 0.01) decreased at follow‐up.

**Conclusions:**

Cohort heterogeneity challenged the effectiveness of a standardised care model, and the evolving nature of Patient Watch complicated impact evaluation. Frequent presenters showed diverse clinical and demographic profiles with high treatment burdens, highlighting the need for tailored care.

## Highlights

**What is already known on this subject?:**

- Case management to support frequent presenters to the ED has been shown to be effective and Acceptable, however the evidence is largely drawn from well‐resourced metropolitan settings.

**What this paper adds?:**

- Provides rural‐specific evidence on the experiences and impact of a case management programme adapted from a metropolitan model to support frequent presenters to the ED.
- Shows that time‐limited interventions may not be sufficient for patients with complex, ongoing needs, highlighting the importance of post‐programme planning and long‐term support that considers rural resource constraints.
- Demonstrates the importance of evaluating patient experiences and outcomes when established interventions are translated beyond their original setting to ensure they remain effective, responsive to the local context and patient‐centred.

## 1 Introduction

Globally, unsustainable demand for emergency department (ED) care [1] is associated with negative effects on patient health outcomes, healthcare staff and healthcare spending [2]. ED crowding has led to a focus on individuals who are considered ‘frequent presenters’, variably defined as those presenting 3–7 times/year [3]. These individuals are often heavy users of the broader healthcare system and have complex physical, mental and social needs [3]. It has been reported that people who present at least 3 times/year represent 3%–10% of all ED patients and account for up to 34% of total ED presentations [4]. Given a significant proportion of ED presentations are considered potentially ‘inappropriate’ (i.e., non‐urgent) presentations that may be addressed in primary care [5], models of care that reduce demand for ED care are warranted.

Case management, a collaborative approach coordinating care to meet patient needs [6], has been frequently implemented to improve care for frequent presenters. Several reviews have reported positive effects from case management with reference to reducing hospital admissions, ED presentations, length‐of‐stay and healthcare costs [7, 8, 9]. Few studies have evaluated the experiences of frequent presenters who receive case management interventions, or their caregivers. The limited evidence suggests frequent presenters perceive case management provides value (e.g., improved health literacy and interactions with healthcare system) [10, 11], but also has negative aspects (e.g., stigmatisation, unclear purpose) [11]. Patient experience is increasingly valued for healthcare quality with positive experiences associated with improved clinical effectiveness, patient safety and less healthcare use [12].

Although case management interventions are increasingly adopted worldwide, most evaluations occur in well‐resourced metropolitan tertiary services with readily available referral options [7, 8, 9]. Scaling case management across rural areas with considerable geographical distances, dispersed populations and workforce shortages requires significant contextual adaptation [13]. As shown in our previous work, this has included greater reliance on telehealth and a shift from multidisciplinary care to nurse‐delivered care [13]. The potential impacts of adaptations required to deliver case management at scale to lower‐resourced rural contexts are unknown, but the potential exists for altered patient experiences and outcomes.

In this study, Patient Watch, a telehealth case management model of care to manage the care of frequent presenters, was evaluated. This study aimed to explore: (1) patients' experiences and perceptions of Patient Watch and (2) the effect of Patient Watch on ED presentations (primary outcome), healthcare use, quality of life and chronic illness care (secondary outcomes).

## 2 Materials and Methods

### 2.1 Study Design

The study design was a prospective longitudinal cohort study with a convergent–parallel mixed methods approach, which allowed for collecting and separately analysing quantitative and qualitative data before converging the interpretation of findings [14]. A mixed methods evaluation was chosen to quantify outcomes and explore patient experiences of Patient Watch to holistically understand how and why things work. Our recent systematic review [15] identified that the likely impact of the programme would depend on the model as it evolved and the characteristics of the patient cohort. Therefore, a pragmatic sample size was chosen to allow exploration of multiple potential outcomes. The study was informed by critical realism, which acknowledges reality beyond what is observable, as well as the roles of human interpretation and social structures in shaping our understanding of reality [16]. Critical realism supports various methodological approaches, including mixed methods, to explain how and why phenomena occur [17].

Patients and carers were not directly involved in the design or conduct of this study. Multiple attempts to recruit them to the steering committee throughout the evaluation were unsuccessful due to the complex and unstable mental and physical health, as well as challenging social circumstances of the target population.

### 2.2 Study Setting

The study was undertaken at a public health service with a catchment population of about 250 000 in Victoria, Australia [18]. The study included participants seeking healthcare across modified Monash model areas 2–5 (i.e., regional centres through to small rural towns) [19]. Several catchment areas had a lower health status and socioeconomic status than Victorian state‐wide averages [18]. Patient Watch was implemented in 2022 to reduce frequent ED presentations.

### 2.3 The Patient Watch Model

Patient Watch has been described in detail previously [13]. In brief, Patient Watch has five core functions: (1) identifying candidates using a predictive algorithm or through referrals, with screening to determine eligibility; (2) inducting eligible candidates into the programme and performing an initial health assessment with the health coach; (3) lay tele‐navigator support (TNS) workers providing monitoring through weekly phone check‐ins supported by a record system providing real‐time risk alerts for adverse changes; (4) ensuring any alerts are followed by a health coach coordinating interventions with community, hospital, or social service providers and (5) conducting six‐weekly reviews with the health coach to determine ongoing need for the programme.

### 2.4 Participants and Recruitment

Participants were eligible for the study if they were enrolled in Patient Watch and aged at least 18 years. Exclusion criteria included limited English language proficiency, inability to provide informed consent (e.g., cognitive impairment), living in residential aged care, a prognosis of less than 1 year or participation in an alternative case management programme.

TNS workers provided a brief overview of the research and sought permission from all eligible Patient Watch candidates to forward potential participants' contact details to researchers. Researchers then sent a letter and plain language statement, followed by a phone call to discuss the study, answer questions and seek consent for participation. Telephone interviews were scheduled at a convenient time, with verbal consent recorded prior to the interview. Participants received an AU$20 gift voucher in recognition of their participation.

### 2.5 Data Collection

Data were collected at baseline (recruitment to Patient Watch) and at follow‐up (6 months post‐recruitment) from several sources:

1. Telephone interviews were conducted at baseline and follow‐up. A structured questionnaire was administered, collecting demographic information, current health issues, general and emergency health service use. Two validated tools were also administered:
  - Assessment of Quality of Life‐8D (AQoL‐8D) (35‐items). This tool measures eight dimensions and two super‐dimensions of physical (independent living, pain, senses) and psychosocial (relationships, mental health, coping, self‐worth, life satisfaction) quality of life, as well as providing an overall score. Each item has 4–6 response levels with utility weights ranging from one (best health) to zero (death) [20];
  - PACIC (20 items) measuring five sub‐scales: activation, decision support, goal setting, problem‐solving and coordination, as well as providing an overall score. Items are scored from 1 to 5, with higher scores reflecting better alignment with the Chronic Care Model [21].
2. Semi‐structured interviews conducted at follow‐up (see Supporting Information) to explore initial engagement, experiences of care received, preparation for exit and suggestions for improvement.
3. Collection of variables including ED attendance (number), arrival transport mode, triage category, primary provisional diagnosis and admissions from the Victorian Emergency Minimum Dataset [22], 6 months prior to baseline and at follow‐up.
4. Patient Watch programme data collected at baseline and follow‐up, including mode of entry (e.g., algorithm or referral), health conditions, programme metrics (e.g., number/duration of calls, escalation of care, discharge status) and the Patient Health Questionnaire for Depression and Anxiety (PHQ‐4) (four‐items). Scores range from 0 to 12: normal (0–2), mild (3–5), moderate (6–8) and severe (9–12) [23].

### 2.6 Data Analysis

#### 2.6.1 Qualitative

The team involved in the analysis included researchers with clinical (pharmacy, physiotherapy) and/or extensive health services and public health research experience. All semi‐structured interviews were conducted, audio‐recorded and transcribed verbatim by the first author (M.M.). Reflexive thematic analysis followed Braun and Clarke's six‐phase approach [24]. Analysis was led by M.M., in collaboration with A.W.S. and K.M.N.

Following transcription, interviews were read and re‐read with initial ideas for coding noted. Analysis combined inductive and deductive approaches, focusing on participant experiences with Patient Watch and remaining open to all possible interpretations. Codes were grouped into initial candidate themes, explored for distinctiveness or overlap and reorganised to better reflect themes. Themes were identified on a semantic and latent level and extracts checked for consistency within each code and theme. QSR NVivo 14 was used to support analysis.

Data collection and analysis were informed by the researchers' prior experience and knowledge of literature and experience with similar health programmes. Reflexivity was part of the approach with post‐interview debriefing sessions, collaborative discussions and reflective notetaking used to interpret findings and ensure findings reflected the data and balanced with individual views.

#### 2.6.2 Quantitative

Statistical analyses were conducted using Stata version 18.0 (StataCorp LP, College Station, Texas, USA). Descriptive statistics included means and standard deviations (for continuous variables) or percentages (for categorical variables). Group comparisons were made using paired *t*‐tests or Wilcoxon signed‐rank tests (continuous variables), and *χ* ^2^ tests (categorical variables). *p* < 0.05 was considered significant. Subgroup analyses explored increased ED presentations at follow‐up, higher acuity presentations at baseline and follow‐up and different entry modes into Patient Watch. Given small sample sizes, subgroup outcomes were by inspection rather than statistical testing.

### 2.7 Convergent Analysis

The quantitative and qualitative findings were compared through collaborative discussion between M.M., A.W.S. and K.M.N. Qualitative themes were mapped to findings from the quantitative analysis to achieve a more complete understanding of the effects of the Patient Watch programme.

## 3 Results

**FIGURE 1:**
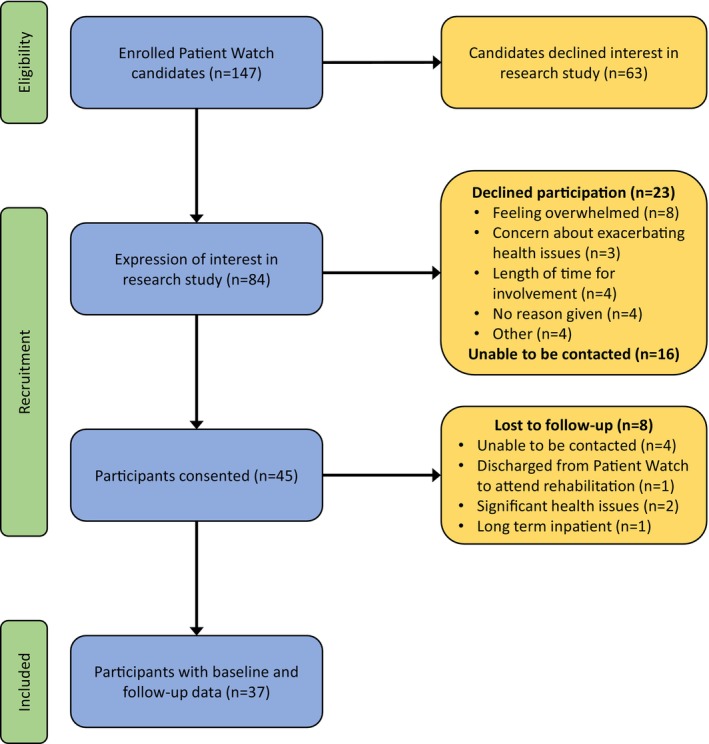
Participant flow through the study.

During the data collection period from August 2023 to May 2024, 147 candidates were enrolled in Patient Watch. Because of incomplete health service records, it was not possible to determine enrolment rates for the programme. Of those enrolled, 84 expressed interest in study participation. Forty‐five (54%) agreed to participate, 23 (27%) declined and 16 (19%) could not be contacted. Reasons for declining participation included feeling overwhelmed (e.g., because of age or hospitalisation), concerns about health or mental health impacts and the time required for participation (Figure 1).

At follow‐up, 37 of 45 participants (82%) completed data collection at both time points. Of those that did not, four could not be contacted, two declined because of significant health issues, one was not approached because of long‐term inpatient status (where Patient Watch care was not provided) and one had been discharged and was in drug and alcohol rehabilitation (Figure 1).

### 3.1 Participant Characteristics

Participant characteristics are reported in Table 1. Participants (*n* = 37) included 20 (54%) females and had a mean age of 71.3 ± 11.8 years. Common presenting health conditions were chronic obstructive pulmonary disease (COPD) (*n* = 10, 27%) and chronic heart failure (*n* = 8, 22%); 15 (41%) participants had a diagnosed mental illness.

**TABLE 1:** Characteristics of participants.

Characteristic	*n* = 37
Age, mean (SD)	71.3 (11.8)
Female, *n* (%)	20 (54)
Education level, *n* (%)	
Primary school	3 (8)
Secondary school	12 (32)
Further education (university/vocational)	22 (60)
Living arrangement, *n* (%)	
Live alone	14 (38)
Partner/spouse	14 (38)
Other (e.g., siblings, share house)	9 (24)
Australian healthcare concession card, *n* (%)	
No concession card	6 (16)
Concession card	31 (84)
Main health condition for programme participation, *n* (%)	
COPD	10 (27)
Heart failure, chronic	8 (22)
Hypertension	5 (14)
Diabetes type 2	2 (5)
Osteoarthritis	2 (5)
Other^a^	10 (27)
Mental health diagnosis, *n* (%)	15 (41)
Number of chronic conditions, mean (SD)	6.2 (2.7)
PHQ4, mean (SD)	3.2 (3.3)
Days between baseline and follow‐up interviews, mean (SD)	161.5 (13.1)
Days enrolled in Patient Watch at follow‐up interview, mean (SD)	173.9 (32.5)

Abbreviations: COPD, chronic obstructive pulmonary disease; PHQ4, Patient Health Questionnaire for Depression and Anxiety.

^a^ Bronchiectasis, lymphoedema, acquired brain injury, pneumonia, schizophrenia, chronic pain, asthma, urinary incontinence and stroke.

### 3.2 Qualitative Results

Four themes were constructed from the experiences and perceptions of Patient Watch 6 months post‐recruitment: (1) experiences with chronic illness and receiving care, (2) barriers to navigation of the health system, (3) positive experiences with Patient Watch care and (4) bridging gaps: more to be done. Whereas Themes 1 and 2 reflect pre‐enrolment experiences and Themes 3 and 4 relate to experiences post‐enrolment.

#### 3.2.1 Theme 1: Experiences With Chronic Illness and Receiving Care

This theme captures participants' experiences of living with chronic illnesses characterised by complex physical, mental and social needs. Many faced multimorbidity, multiple care needs and were served by multiple healthcare providers. This affected daily functioning physically (e.g., persistent pain, medication side effects), psychologically (e.g., reduced confidence, anxiety), socially (e.g., strained relationships, increased dependency on family), financially (e.g., difficulties affording rent or healthcare) and recreationally (e.g., inability to engage in leisure activities). These challenges caused stress and significant unmet health, psychological and social needs, exacerbating distress:

You know what is my life about? Constant pain, struggling with finances, struggling to keep a roof over my head. (ID26, female, 69 years)

Unexpected health changes or acute deterioration increased healthcare use, resulting in a cascade of appointments, assessments and follow‐up care with multiple doctors across several health services. Lack of continuity of care, both in hospital and during transitions, was a common concern, with confusion over care coordination and accountability:

In fact, one of the problems that I found … when you're in the hospital … every day it seems to be a different doctor, you know, and there's no continuation from day‐to‐day…. (ID2, male, 86 years)

Participants reported poor communication between health professionals and across services, leading to repeated recounting of medical histories, conflicting or insufficient information and referral delays. Participants also perceived a lack of patient‐centred care with decisions made without consultation (e.g., medication changes), limited treatment options and strict adherence to standard clinical pathways without considering individual needs:

I'm not knocking the doctors and nurses. They're brilliant, they're good, but they're just, they're just following, they just seem to be following a procedure… (ID2, male, 86 years)

Participants also described experiences of misdiagnosis, inappropriate (e.g., changes in medication) or delayed care (e.g., lack of timely referral to specialists) and a lack of follow‐up (e.g., discharged from ED without diagnosis or plan), which contributed to further distress and avoidance of doctors, hospitals and the ED:

… he said I'm specialist so and so. Two minutes. Two minutes. He said, yeah, all your bloods are OK. Yeah, can't see what the problem is. Yep, you can go home. If it happens again, just give us a ring. And I thought, what, do you think I'm going to ring and lay down here [at the emergency department] for seven hours [until] you tell me I've taken your bloods, everything looks fine. (ID30, male, 74 years)

#### 3.2.2 Theme 2: Barriers and Delays With Navigation of the Health System

This theme describes the perceived barriers and delays in accessing healthcare before Patient Watch enrolment. Participants noted health system strain with increased demand (e.g., ambulance delays, hospital bed shortages, long ED wait times):

There was four of us, all heart patients, took us out of the corridor, took us into the general waiting bay [of the emergency department]. In the 5 1/2 hours I was there, two slid out of their chairs because they were in so much pain. (ID30, male, 74 years)

Long waiting times for appointments, short consultations and referral delays to specialists further hindered access. Affordability was another concern because of the gap fee or out‐of‐pocket costs associated with GPs, specialists and clinical tests:

I was seeing a lung specialist, but I only went once. I couldn't afford to keep going and I'd said to my doctor about not being able to afford it, can he put me through the [public] hospital so that I can … go to there instead of going to a private one. (ID34, female 74 years)

Rising costs for private health insurance led some to cancel coverage. Participants also perceived there was inadequate funding to extend support from time‐limited services (e.g., Hospital Admissions Risk Program) or to pay for clinical tests through Medicare, Australia's universal health insurance scheme. Applying for My Aged Care (MAC), a federal government scheme to support home‐based care for older adults, was described as challenging, with long waits and provider shortages, especially in rural areas:

I was, from [My] Aged Care, I was allocated someone to help me out and not only have they not come, but they've got no one that can do it. So, there's no providers in [the regional centre] that can do it. (ID44, male, 65 years)

#### 3.2.3 Theme 3: Positive Experiences With Patient Watch Care

This theme outlines key elements of Patient Watch: collaborative care planning, health monitoring and tailored actions. Although many participants did not recall developing a care plan, some identified its purpose as setting goals to improve health and avoid hospital or ED presentations. Participants suggested Patient Watch monitored their health, supported goal adherence, provided reminders for appointment attendance and medications, and prompted additional health coach support as needed:

It's just what I expected, somebody [tele‐navigator support worker] to call, check in, see how I'm going, remind me of appointments if I've got them…. (ID12, female, 69 years)

Participants reported health coaches coordinated referrals to GPs, specialists and allied health professionals (e.g., psychologists, dieticians, physiotherapists), scheduled appointments, suggested referrals, advocated for services such as MAC, arranged short‐term in‐home support (e.g., personal care support, pathology collection) and offered regular reminders for medications and appointments:

I had a reference to uh, some psychological support which I needed at the time, which is absolutely wonderful. Fantastic. Best thing I ever did. (ID14, male, 61 years)

[My Aged Care] said, oh, another 12 to 15 months. [The health coach] got onto them, [support was approved in] three weeks. (ID30, male, 75 years)

These actions offered reassurance and a sense of accessible support during times of need. Health coaches provided informational support (e.g., knowledge and facts about health and/or specific conditions):

… [health coach] also sends out literature for me that I need and then she'll go through it with me on the phone to make sure that I'm doing, using puffers right or, you know, cleaning and properly and having everything at the right time. (ID34, female, 74 years)

Health coaches also delivered esteem‐building support (e.g., affirmation, motivation and confidence) and actively promoted self‐management:

Well, they're advising me that no, you know, don't need to go to the hospital for that. You can go to the doctor. That's fine or no, you really need to go in and see the emergency department it's a serious thing. (ID32, female, 49 years)

Further, health coaches offered both emotional support (e.g., care and comfort) and practical assistance (e.g., arranging appointments, validating actions to take). Participants felt that Patient Watch supported shared decision‐making, with health coaches helping to prevent acute health issues and improving carer burden, psychological wellbeing (e.g., reduced anxiety) and confidence:

Yeah, and peace of mind, I guess is the other thing that was important just to know that I was on the right track…. (ID16, male, 68 years)

For many socially isolated participants, regular contact also provided a meaningful connection, often serving as their only consistent social interaction:

Well, it's a bit of touching base with somebody [at Patient Watch] because some days I don't get phone calls from anybody. It's nice to have somebody to ring, to ring to find out how you are. My family doesn't ring me very often and it's nice to have somebody. (ID22, female, 85 years)

#### 3.2.4 Theme 4: Bridging the Gaps: More to Be Done

This theme describes the gaps perceived by participants between their expectations and experiences of Patient Watch. Although many spoke positively about the programme, confusion about the programme's purpose, recruitment process and components (e.g., care planning, monitoring, tailored actions) was common. Some only understood its goal of reducing hospital and ED presentations after multiple phone calls:

I didn't even know what it [Patient Watch] was all about, to be honest with you. It was all new to me for the first few calls, I thought why do they keep ringing me for until I worked it all out. (ID29, male, 61 years)

For several participants, the benefits of Patient Watch were unclear. In contrast to the reassurance and connection it offered some (Theme 3), for others, weekly monitoring phone calls were often viewed as repetitive and low value, providing no medical advice beyond escalation to a health coach:

I mean, the weekly calls fine. It's just, I don't think there's much point in it … because, I'm just saying, I'm just telling them [TNS worker] the same stuff every week. (ID9, male, 42 years)

Health coach actions were sometimes seen as superficial or redundant with an overreliance on GP referrals, which participants already accessed:

A couple, a couple of times I needed, a nurse phoned me and suggested I see a GP. So, in terms of that, well, if it was bad enough I would anyway. So, I just don't find, I like the monitoring only, but I find that, the medical side isn't really helpful. (ID26, female, 69 years)

Some felt their complex needs exceeded the scope of the programme and suggested other programmes (e.g., Hospital Risk Admissions Program) offered more comprehensive, face‐to‐face care and better links to health and social services. Although few suggested improvements and some saw no immediate benefit, many felt the programme could be valuable long‐term if their health declined. Participants were reassured that Patient Watch remained easily accessible at any time:

I get reinforced that you got our [Patient Watch] numbers … and I've got it on my contacts list on the phone, so if I, I've got all the support numbers, if I need it. (ID24)

Suggestions for improvement included stronger advocacy for service access (e.g., specialists, MAC), more thorough assessment of health declines, education on available health and social services available and support for social needs such as financial hardship:

So, I suppose a bit more of an understanding, it's not only medical, it's also about the state of life, you know, where I am at and what's worrying me the most sort of thing. (ID26, female, 69 years)

### 3.3 Quantitative Results

#### 3.3.1 Clinical Outcomes

Table 2 presents the analysis of ED data, self‐reported healthcare use—including the AQoL‐8D and PACIC—comparing baseline to follow‐up. Significant reductions were observed in ED presentations (2.2 ± 2.1 vs. 1.0 ± 1.9, *p* < 0.001), hospital admissions (1.5 ± 1.0 vs. 0.5 ± 0.9, *p* < 0.001), GP visits (8.1 ± 5.6 vs. 6.8 ± 6.7, *p* = 0.02) and specialist visits (3.6 ± 4.6 vs. 2.1 ± 2.4, *p* = 0.01). No significant changes were observed in AQoL‐8D or PACIC scores.

**TABLE 2:** Clinical outcomes pre‐ and post‐Patient Watch.

	Baseline (*n* = 37)	Follow‐up (*n* = 37)	*p*
*Emergency department data*	*n* = 77^a^	*n* = 36^a^	
ED presentations, mean (SD)	2.2 (2.1)	1.0 (1.9)	< 0.001
Triage category, *n* (%)			
Resuscitation	0	1 (3)	0.46
Emergency	21 (27)	10 (28)	
Urgent	37 (48)	19 (53)	
Semi‐urgent	18 (24)	5 (14)	
Non‐urgent	1 (1)	1 (3)	
Number of admissions, mean (SD)	1.5 (1.0)	0.5 (0.9)	< 0.001
*Self‐reported data*			
Number of GP visits, mean (SD)	8.1 (5.6)	6.8 (6.7)	0.02
Number of specialist clinician visits, mean (SD)	3.6 (4.6)	2.1 (2.4)	0.01
AQol‐8D, mean (SD)			
Independent living domain	0.67 (0.19)	0.65 (0.18)	0.15
Happiness dimension	0.71 (0.16)	0.71 (0.15)	0.92
Mental health dimension	0.60 (0.12)	0.61 (0.13)	0.67
Coping dimension	0.70 (0.16)	0.71 (0.15)	0.46
Relationships dimension	0.65 (0.15)	0.65 (0.14)	0.75
Self‐worth dimension	0.69 (0.18)	0.72 (0.15)	0.20
Pain dimension	0.57 (0.27)	0.54 (0.28)	0.46
Senses dimension	0.85 (0.12)	0.85 (0.10)	0.81
Super‐dimension: mental	0.30 (0.17)	0.30 (0.15)	0.99
Super‐dimension: physical	0.50 (0.20)	0.48 (0.21)	0.39
Utility score	0.57 (0.20)	0.57 (0.19)	1.00
PACIC, mean (SD)			
Patient activation	3.2 (1.4)	3.2 (1.5)	0.97
Delivery system design/decision support	3.8 (1.9)	3.5 (1.3)	0.16
Goal setting/tailoring	3.4 (1.3)	3.3 (1.4)	0.56
Problem‐solving/contextual counselling	3.1 (1.3)	3.3 (1.5)	0.47
Follow‐up/coordination	3.1 (1.2)	2.8 (1.3)	0.15
Total	3.3 (1.2)	3.2 (1.2)	0.76

Abbreviations: AQoL‐8D, Assessment of Quality of Life‐8D; GP, general practitioner; PACIC, Patient Assessment of Chronic Illness Care.

^a^ Total emergency department presentations.

#### 3.3.2 Patient Watch Programme Data

Table 3 presents Patient Watch programme data. Most participants were referred (*n* = 24, 65%), with fewer identified via the HLCC algorithm. Participants received 15.5 ± 5.8 TNS worker calls with a mean 13.6 ± 5.4 min duration and 7.5 ± 3.6 HC calls with a mean duration 58.6 ± 22.1 min. At 6‐months post‐enrolment, 28 (76%) participants remained enrolled in Patient Watch.

**TABLE 3:** Patient Watch programme data.

	*n* = 37
Mode of entry, *n* (%)	
Referral	24 (65)
HLCC algorithm	13 (35)
TNS worker, mean (SD)	
Number of calls	15.5 (5.8)
Duration per call (min)	13.6 (5.4)
Did not answer	4.0 (3.5)
Health coach, mean (SD)	
Number of calls	7.5 (3.6)
Duration per call (min)	58.6 (22.1)
Did not answer	1.2 (2.3)
Problems and actions, mean (SD)	1.9 (2.4)
Status at follow‐up, *n* (%)	
Continuing	28 (76)
Discharged	9 (24)

Abbreviation: TNS, tele‐navigator support.

#### 3.3.3 Emergency Department Presentations

Within this cohort, 30 participants had fewer ED presentations and seven participants had more ED presentations at follow‐up compared with the 6‐month period prior to enrolment (see Tables S1–S3). Although there were insufficient sample sizes for statistical analysis, participants with more ED presentations tended to be male (71% vs. 50%), were less likely to have a mental health diagnosis (29% vs. 43%) and had lower PHQ4 scores (1.4 vs. 3.6). They also had more admissions (1.6 vs. 0.2) and lower PACIC scores (2.5 vs. 3.4) at follow‐up compared with the baseline. Further, they received more health coach calls (9.1 vs. 7.1), had more problems/actions raised (3.9 vs. 1.4) and were less often discharged from Patient Watch (14% v. 27%).

#### 3.3.4 Emergency Department Triage Categories

Notable differences were observed between participants with higher acuity (resuscitation/emergency/urgent, *n* = 10) and lower acuity (semi‐urgent/non‐urgent, *n* = 27) ED presentations at baseline (see Tables S4–S6). Although the sample size limited statistical analysis, the higher acuity group more often had a mental health diagnosis (50% vs. 37%) and higher PHQ4 scores (4.6 vs. 2.7). They also had more ED presentations (4.1 vs. 1.4), GP visits (11.4 vs. 6.8), specialist visits (4.7 vs. 3.2) and lower AQoL‐8D utility scores (0.45 vs. 0.61). They received more TNS worker calls (18.3 vs. 14.5), with longer average call durations (17.0 vs. 12.3) and were less frequently discharged from Patient Watch (10% vs. 29%). Of the higher acuity group, three remained in that category at follow‐up along with one participant who shifted into this group. At follow‐up, higher acuity patients showed similar patterns, including higher rates of higher mental health diagnoses, greater health service use, lower quality of life scores and continued programme engagement compared with lower acuity patients.

#### 3.3.5 Mode of Entry Into Patient Watch

Differences were observed between participants referred to Patient Watch (*n* = 24) and those identified by the HLCC algorithm (*n* = 13) (see Tables S7–S9). Although the sample size was too small for statistical analysis, the HLCC algorithm‐identified participants were more often male (69% vs. 46%), had fewer chronic conditions (5.2 vs. 6.7) and higher PHQ4 scores (4.0 vs. 2.8). They had more ED presentations (BL: 2.9 vs. 1.8; FU: 1.4 vs. 0.8), admissions (BL: 2.2 vs. 1.0; FU: 0.6 vs. 0.4) and GP visits (BL: 9.1 vs. 7.5; FU: 8.1 vs. 6.1) at baseline and follow‐up. They received fewer TNS worker (13.8 vs. 16.5) and health coach (6.1 vs. 8.2) calls, raised fewer problems/actions (1.3 vs. 2.2) and were discharged more often (*n* = 5, 39% vs. *n* = 4, 17%).

### 3.4 Synthesis

The inferences of the two datasets are represented by the integration of the mixed methods shown in Table 4.

**TABLE 4:** Synthesis of qualitative and quantitative analysis.

Proposition	Study findings from qualitative component	Study findings from quantitative component
High patient complexity, declines in physical, psychological and emotional functioning, as well as social vulnerability may contribute to negative healthcare experiences and distress from care received.	Participants described their experiences of living with complex, chronic illness and receiving healthcare. There were descriptions of declines in physical, psychological and emotional functioning, loss of social connections, negative healthcare experiences and how they were all connected.	At baseline, this cohort had multiple chronic conditions with mental health comorbidities and low quality of life scores. They also had a PHQ‐4 score indicative of mild psychological distress and characteristics of social vulnerability (e.g., concession card holders, living alone and lower educational attainment).
The healthcare system has limited capacity to meet the needs of this at‐risk cohort of individuals with complex healthcare needs. Challenges relate to the accessibility of care and the navigation of care.	Participants reported challenges with accessibility of care and healthcare system navigation, as well as with timeliness and duration of appointments, affordability and difficulties with MAC prior to Patient Watch enrolment.	At baseline, this cohort had complex healthcare needs and high levels of healthcare use across multiple levels of healthcare. PACIC scores were low, particularly for the subscale follow‐up/coordination, suggesting a need for healthcare system navigation.
Patient Watch helped to support healthcare needs by coordinating care and facilitating healthcare access, helping patients manage acute exacerbations of chronic disease. It may contribute to decreased healthcare use.	Participants described that Patient Watch helped support their health needs and coordinated care by establishing a care plan, managing transitions of care, enabling patient engagement with actionable information and facilitating access to timely healthcare. Care coordination was mediated through informational, emotional, esteem‐building and practicable support.	There was a statistically significant decrease in ED presentations, hospital admissions, GP visits and specialist clinician visits. ED presentations were less likely to be triaged urgent/semi‐urgent (not significant), suggesting reduced unnecessary use of ED.
Most participants remained with Patient Watch 6 months post‐enrolment, reflecting the presence of ongoing or anticipated needs.	Participants suggested that although Patient Watch had potential, its impact could be enhanced by including more extensive referral pathways beyond GPs, advocacy with healthcare services to support care needs, a deeper exploration of health declines, education about and linkages to appropriate community services and a greater focus on social issues (e.g., financial).	A high proportion of participants continue with the Patient Watch 6 months post‐enrolment, with no change in quality of life or PACIC scores. Different subgroups of frequent presenters with distinct characteristics and outcomes were identified (e.g., increased ED presentations at follow‐up, presenting to the ED with higher acuity presentations at baseline and follow‐up, mode of entry into Patient Watch).

## 4 Discussion

This study provides valuable evidence for the impact of a rural telehealth programme implemented using a case management model of care to manage the care of people who frequently present to the ED. The study provides a novel and in‐depth analysis of participant experiences within Patient Watch in a rural context in Australia. Findings suggest that Patient Watch supports frequent presenters by coordinating care and facilitating access to healthcare services, support that may decrease healthcare use. Our analysis also revealed clinical and demographic heterogeneity among frequent presenters, suggesting a need for the tailoring of the programme to effectively address presenters' unique needs.

Although the study found case management is valued with some gains, the treatment burden remains high and continued support for self‐management is needed. The reported lack of improvement in chronic illness care and quality of life may be because, typically, evaluations of interventions to address frequent presentations refer to averages or changes in averages [7, 8, 9], largely ignoring individual variability. Despite several reviews highlighting the heterogeneity of frequent presenters [7, 8, 9] and recent studies identifying subgroups with differing characteristics and risk profiles [25, 26, 27], few studies have investigated differences in individual responses to case management. Our results show that the treatment burden remains high for some subgroups of frequent presenters and that these participants may need longer for engagement, self‐management and improvement to their overall health. Understanding the characteristics that determine why some frequent presenters respond better to case management than others may help to uncover important differences within the whole population that should be considered when evaluating a universal case management intervention.

The prolonged enrolment of our participants raises questions about the feasibility of delivering the programme as a time‐limited intervention. Although time‐bound models can support resource allocation and scalability, they may be less suited to individuals with complex, ongoing needs. A finite model requires clearly defined transition and disengagement planning to ensure continuity of care, including preparing participants for discharge, linkage to appropriate follow‐up services and ensuring adequate support remains in place. Evidence from similar programmes suggests that post‐programme planning is often overlooked, with limited consideration given to the availability of ongoing support or the capacity of the healthcare system to maintain the health gains achieved during programme participation [15, 28]. Long‐term outcomes—including health status, healthcare use or quality of life 1 year after programme discharge—are rarely tracked, limiting understanding of sustained impact [28]. These challenges may be exacerbated in rural contexts, where service access is constrained by workforce shortages, geographic barriers and limited infrastructure [29, 30, 31]. For high‐need individuals, programme exit may result in significant care gaps, highlighting the importance of tailored discharge strategies and integrated healthcare service planning that account for both patient needs and local resource limitations.

Compared with previous research, the diversity of patient experiences in this study may well reflect the rural context for implementation. As previously identified, Patient Watch evolved from its metropolitan‐centric origins through necessity to sustain the programme's financial viability while also accommodating the need to deliver services across greater distances, with fewer resources and to more dispersed populations [13]. This entailed shifting from face‐to‐face case management towards a telehealth monitoring and advisory service and expanding the target population [13]. Patient Watch, consequently, became a region‐wide resource for people with limited options, serving a broader, heterogeneous population compared with that in metropolitan interventions, which often focus on more narrowly defined populations [15, 32, 33]. The increased heterogeneity of both patient needs and available referral options arising from this context may add to the challenge of optimising patient experiences and meeting all healthcare needs in rural case management.

Evaluating such interventions as Patient Watch in dynamic real‐world settings is challenging. Given the diverse and changing needs of participants, and the adaptive nature of Patient Watch, we caution against relying on conventional outcomes (e.g., ED presentations) to assess impact. Although RCTs are considered the gold standard for assessing efficacy, they often fail to capture the complexity of long‐term, individualised interventions within dynamic contexts [34]. Our evaluation, which is grounded in multiple perspectives, including lived experience, has provided a nuanced understanding of its impact. For example, we identified multiple layers of complexity, with the health system (e.g., care silos, fragmentation), as well as medical (e.g., multimorbidity, care needs) and situational (e.g., patient circumstances) factors often hindered effective care prior to enrolment. Whereas Patient Watch was found to be beneficial from multiple perspectives, objective data alone did not fully capture its impact. These findings suggest the need for evaluations that consider appropriate, individualised outcomes and recognise case management as a flexible, integrated approach rather than one restricted to a specific population. Although reductions in ED presentations and admissions suggest the potential for healthcare cost savings, a formal economic evaluation was beyond the scope of the current study. Future research should include an economic evaluation to assess the costs of programme delivery along with potential savings associated with reduced healthcare use.

### 4.1 Strengths and Limitations

A key strength of this study includes the mixed methods approach that obtains diverse perspectives from which a comprehensive understanding of a complex intervention can be established. Limitations include the small sample size for the quantitative component, which limited the power of our analyses and ability to explore variability in response to Patient Watch. A further limitation is the lack of a control group for the quantitative phase, which means we cannot rule out that health service use would have become less frequent over time (i.e., regression‐to‐the‐mean), even without intervention. However, a mixed methods approach identifies mechanisms by which Patient Watch may be helping. Another limitation was the low participant uptake in the evaluation, raising uncertainty about the representativeness of the quantitative data. This study was also conducted at a single rural health service which may not be representative of issues in other rural settings. Finally, limited engagement of patients and carers on the steering committee restricted opportunities to inform care model development, study design, evaluation and interpretation of findings.

## 5 Conclusions

Our study suggests Patient Watch supported frequent presenters and may have helped reduce healthcare use in a rural context. Patient Watch served a heterogenous population with distinct subgroups differing in needs, behaviours and outcomes, highlighting the importance of tailoring such programmes to address differences effectively. Although evaluating Patient Watch was challenging, given its evolving nature, incorporating multiple perspectives—including lived experiences—offered a nuanced understanding of the programme's impact and the diverse realities of participants. Our findings suggest evaluations should consider individualised outcomes and tailor discharge planning aligned with patient needs and local resources.

## Author Contributions

**Pheona Griffith:** resources, validation, writing – review and editing. **Mary Malakellis:** data curation, formal analysis, methodology, writing – original draft, writing – review and editing, validation. **Jade Odgers:** resources, writing – review and editing. **Kevin Mc Namara:** formal analysis, investigation, methodology, supervision, validation, writing – review and editing. **Vincent L. Versace:** supervision, writing – review and editing. **Laura Alston:** supervision, writing – review and editing. **Anna Wong Shee:** formal analysis, investigation, methodology, project administration, supervision, writing – review and editing, validation.

## Disclosure

This paper is original and has not been published elsewhere, nor is it currently under consideration for publication elsewhere. The authors declare that no artificial intelligence (AI), large language models (LLMs) or generative AI tools were used in the research, data analysis or preparation of this manuscript.

## Ethics Statement

Ethics approval was received from the Deakin University Health Ethics Advisory Group (2021‐444) and the Ballarat Health Services and St John of God Hospital Human Research Ethics Committee (HREC/78891/BHSSJOG‐2022‐317489(v3)).

## Conflicts of Interest

The authors declare no conflicts of interest.

## Supporting information


**Table S1:** Characteristics of participants.
**Table S2:** Clinical outcomes pre‐ and post‐patient watch.
**Table S3:** Patient Watch programme data.
**Table S4:** Characteristics of participants.
**Table S5:** Clinical outcomes pre‐ and post‐patient watch.
**Table S6:** Patient Watch programme data.
**Table S7:** Characteristics of participants.
**Table S8:** Clinical outcomes pre‐ and post‐patient watch.
**Table S9:** Patient Watch programme data.

## Data Availability

The data that support the findings of this study are available on request from the corresponding author. The data are not publicly available due to privacy or ethical restrictions.
