# Supplementary material for: Experiences and Perceptions of Patient Watch, a Rural Telehealth Case Management Model, for Frequent Presenters to the Emergency Department: A Longitudinal Mixed Methods Study
Source: Aust J Rural Health. 2026 Jul 11;34(4):e70232. doi: 10.1111/ajr.70232 (PMC13355087; doi:10.1111/ajr.70232)
Supplement: Supplementary file 1 — Table S1: Characteristics of participants. Table S2: Clinical outcomes pre‐ and post‐patient watch. Table S3: Patient Watch programme data. Table S4: Characteristics of participants. Table S5: Clinical outcomes pre‐ and post‐patient watch. Table S6: Patient Watch programme data. Table S7: Characteristics of participants. Table S8: Clinical outcomes pre‐ and post‐patient watch. Table S9: Patient Watch programme data. [file AJR-34-0-s001.docx]

**Supplementary Materials**

*Semi-structured interview schedule*

1. Are you still receiving care from the Grampians Watch program?

How were you initially engaged by the Grampians Watch program?

Prompt 1: How were you identified for the GW program?

Prompt 2: How was a care plan developed by GW for you?

(Note: Confirm screening, assessment activities, conversations)

Prompt 3: What was important to you for GW to address?

Prompt 4: How were your goals developed?

Please tell us about your experiences with the Grampians Watch program?

Prompt 1: What did you need from the GW program?

Prompt 2: What did the GW program provide for you?

Prompt 3: How were your needs/goals met by the GW program? How did this help with your health management (burden, quality of care, service use, outcomes etc.)

Prompt 4: Are there any issues the GW program did not or could not address?

Prompt 5: How were your expectations met/not met by the GW program?

Prompt 6: How easy or difficult was it for you to participate in the GW program?

Please tell us about how you have been prepared to/expect to exit the Grampians Watch program?

Prompt 1: Tell us what the nurse (health coach) check on when they call you for reviews?

Prompt 2: What would happen if you came off the GW program?

Prompt 3: What support do you need into the future to maintain benefits?

Prompt 4: (If discharged) How were you discharged from the GW program?

Prompt 5: What was the long-term impact of participating in the GW program?

What could be improved about the GW program?

**Emergency department presentations**

**Table S1: Characteristics of Participants**

|  | Increased ED presentations, n = 7 | Decreased ED presentations, n = 30 |
| --- | --- | --- |
| Age, mean (SD) | 75.0 (11.8) | 70.4 (11.9) |
| Male, n (%) | 5 (71.4) | 15 (50.0) |
| Education level, n (%) |  |  |
| Primary school | 1 (14.3) | 2 (7) |
| Secondary school | 3 (42.9) | 9 (30) |
| Further education (university/vocational) | 3 (42.9) | 19 (63) |
| Living arrangement, n (%) |  |  |
| Live alone | 3 (42.9) | 11 (37) |
| Partner/spouse | 3 (42.9) | 11 (37) |
| Other (e.g., siblings, share house) | 1 (14.3) | 8 (27) |
| Australian healthcare concession card, n (%) |  |  |
| No concession card | 0 | 6 (20) |
| Concession card | 7 (100) | 24 (80) |
| Main health condition for program participation, n (%) |  |  |
| COPD^a^ | 3 (43) | 7 (23) |
| Heart failure, chronic | 2 (29) | 6 (20) |
| Hypertension | 0 | 5 (17) |
| Diabetes Type 2 | 0 | 2 (7) |
| Osteoarthritis | 0 | 2 (7) |
| Other^b^ | 2 (29) | 8 (27) |
| Mental health diagnosis, n (%) | 2 (29) | 13 (43) |
| Number of chronic conditions, mean (SD) | 7.1 (2.8) | 6.0 (2.7) |
| PHQ4^c^, mean (SD) | 1.4 (1.3) | 3.6 (3.5) |

^a^ COPD: Chronic Obstructive Pulmonary Disease; ^b^ Other: Bronchiectasis, lymphoedema, acquired brain injury, pneumonia, schizophrenia, chronic pain, asthma, urinary incontinence, stroke; ^c^ PHQ4: Patient Health Questionnaire for Depression and Anxiety.

**Table S2: Clinical Outcomes Pre- and Post-Patient Watch**

|  | Increased ED presentations (n = 7) | | Decreased ED presentations (n = 30) | |
| --- | --- | --- | --- | --- |
|  | Baseline | Follow-up | Baseline | Follow-up |
| *Emergency department data* | *n = 8^a^* | *n = 18^a^* | *n = 69^a^* | *n = 18^a^* |
| ED presentations, mean (SD) | 1.1 (1.2) | 2.6 (1.0) | 2.5 (2.2) | 0.6 (1.9) |
| Triage category, n (%) |  |  |  |  |
| Resuscitation | 0 | 1 (6) | 0 | 0 |
| Emergency | 4 (50) | 9 (50) | 17 (25) | 1 (6) |
| Urgent | 4 (50) | 7 (39) | 33 (48) | 12 (67) |
| Semi-urgent | 0 | 1 (6) | 18 (26) | 4 (22) |
| Non-urgent | 0 | 0 | 1 (2) | 1 (6) |
| Number of admissions, mean (SD) | 1.0 (1.3) | 1.6 (1.1) | 1.6 (0.9) | 0.2 (0.6) |
|  |  |  |  |  |
| *Self-reported data* |  |  |  |  |
| Number of GP^b^ visits, mean (SD) | 10.0 (7.3) | 6.9 (4.3) | 7.6 (5.1) | 6.8 (7.2) |
| Number of specialist clinician visits, mean (SD) | 3.3 (3.3) | 2.1 (1.9) | 3.7 (4.9) | 2.1 (2.5) |
| AQol-8D^c^, mean (SD) |  |  |  |  |
| Independent living domain | 0.72 (0.13) | 0.60 (0.18) | 0.66 (0.20) | 0.66 (0.18) |
| Happiness dimension | 0.70 (0.16) | 0.66 (0.18) | 0.71 (0.16) | 0.72 (0.13) |
| Mental health dimension | 0.60 (0.08) | 0.60 (0.11) | 0.60 (0.13) | 0.61 (0.13) |
| Coping dimension | 0.68 (0.14) | 0.67 (0.15) | 0.71 (0.17) | 0.72 (0.15) |
| Relationships dimension | 0.64 (0.14) | 0.66 (0.17) | 0.65 (0.15) | 0.64 (0.14) |
| Self-worth dimension | 0.71 (0.19) | 0.71 (0.13) | 0.69 (0.18) | 0.73 (0.15) |
| Pain dimension | 0.51 (0.23) | 0.56 (0.29) | 0.58 (0.28) | 0.54 (0.29) |
| Senses dimension | 0.78 (0.13) | 0.78 (0.12) | 0.87 (0.11) | 0.87 (0.10) |
| Super-dimension: mental | 0.29 (0.14) | 0.28 (0.15) | 0.31 (0.18) | 0.31 (0.16) |
| Super-dimension: physical | 0.45 (0.16) | 0.45 (0.21) | 0.51 (0.21) | 0.49 (0.22) |
| Utility score | 0.55 (0.19) | 0.54 (0.22) | 0.58 (0.21) | 0.58 (0.19) |
| PACIC^d^, mean (SD) |  |  |  |  |
| Patient activation | 3.2 (1.8) | 2.4 (1.7) | 3.2 (1.3) | 3.4 (1.4) |
| Delivery system design/decision support | 4.0 (1.0) | 2.8 (1.4) | 3.7 (1.2) | 3.6 (1.2) |
| Goal setting/tailoring | 3.4 (1.6) | 2.2 (1.4) | 3.4 (1.3) | 3.6 (1.2) |
| Problem-solving/contextual counselling | 3.3 (1.3) | 2.6 (1.3) | 3.1 (1.4) | 3.5 (1.5) |
| Follow-up/coordination | 2.9 (1.1) | 2.4 (1.2) | 3.1 (1.2) | 2.9 (1.3) |
| Total | 3.3 (1.3) | 2.5 (1.3) | 3.3 (1.1) | 3.4 (1.2) |

^a^ Total emergency department presentations; ^b^ GP: General Practitioner; ^c^ AQoL-8D: Assessment of Quality of Life-8D; ^d^ PACIC: Patient Assessment of Chronic Illness Care.

**Table S3: Patient Watch Program Data**

|  | Increased baseline ED presentations, n = 7 | Decreased follow-up ED presentations, n = 30 |
| --- | --- | --- |
| Mode of entry, n (%) |  |  |
| Referral | 5 (71) | 19 (63) |
| HLCC Extract (algorithm) | 2 (29) | 11 (27) |
| TNS^a^ worker, mean (SD) |  |  |
| Number of calls | 14.3 (4.6) | 15.8 (6.1) |
| Duration per call (minutes) | 13.9 (2.3) | 13.5 (5.7)) |
| Did not answer | 4.0 (3.0) | 3.9 (3.7) |
| Health coach, mean (SD) |  |  |
| Number of calls | 9.1 (3.8) | 7.1 (3.5) |
| Duration per call (minutes) | 52.4 (24.1) | 60.1 (21.8) |
| Did not answer | 1.7 (1.1) | 1.1 (2.5) |
| Problems and actions, mean (SD) | 3.9 (3.4) | 1.4 (1.9) |
| Status at follow-up, n (%) |  |  |
| Continuing | 6 (86) | 22 (73) |
| Discharge | 1 (14) | 8 (27) |

^a^ Tele-navigator support.

**Lower urgency triaged emergency department presentations**

**Table S4: Characteristics of Participants**

|  | Baseline | | Follow-up | |
| --- | --- | --- | --- | --- |
|  | Triage Category (resuscitation/ emergency/ urgent) (n = 10) | Triage Category (semi-urgent/non-urgent) (n = 27) | Triage Category (resuscitation/ emergency/ urgent) (n = 4) | Triage Category (semi-urgent/non-urgent) (n = 33) |
| Age, mean (SD) | 71.0 (12.6) | 71.4 (11.7) | 68.0 (13.9) | 71.7 (11.7) |
| Male, n (%) | 4 (40) | 16 (59) | 2 (50) | 18 (55) |
| Education level, n (%) |  |  |  |  |
| Primary school | 1 (10) | 2 (7) | 1 (25) | 2 (6) |
| Secondary school | 4 (40) | 8 (30) | 1 (25) | 11 (33) |
| Further education (university/vocational) | 5 (50) | 17 (63) | 2 (50) | 20 (61) |
| Living arrangement, n (%) |  |  |  |  |
| Live alone | 3 (30) | 11 (41) | 0 | 14 (42) |
| Partner/spouse | 4 (40) | 10 (37) | 3 (75) | 11 (33) |
| Other (e.g., siblings, share house) | 3 (30) | 6 (22) | 1 (25) | 8 (24) |
| Australian healthcare concession card, n (%) |  |  |  |  |
| No concession card | 0 | 6 (22) | 0 | 6 (18) |
| Concession card | 10 (100) | 21 (78) | 4 (100) | 27 (82) |
| Main health condition for program participation, n (%) |  |  |  |  |
| COPD^a^ | 3 (30) | 7 (26) | 1 (25) | 9 (27) |
| Heart failure, chronic | 1 (10) | 7 (26) | 0 | 8 (24) |
| Hypertension | 2 (20) | 3 (11) | 1 (25) | 4 (12) |
| Diabetes Type 2 | 0 | 2 (7) | 0 | 2 (6) |
| Osteoarthritis | 0 | 2 (7) | 0 | 2 (6) |
| Other^b^ | 4 (40) | 6 (22) | 2 (50) | 8 (24) |
| Mental health diagnosis, n (%) | 5 (50) | 10 (37) | 2 (50) | 13 (39) |
| Number of chronic conditions, mean (SD) | 7.4 (2.2) | 5.7 (2.8) | 4.8 (2.9) | 6.4 (2.7) |
| PHQ4^c^, mean (SD) | 4.6 (3.7) | 2.7 (3.1) | 5.5 (2.6) | 2.9 (3.3) |

^a^ COPD: Chronic Obstructive Pulmonary Disease; ^b^ Other: Bronchiectasis, lymphoedema, acquired brain injury, pneumonia, schizophrenia, chronic pain, asthma, urinary incontinence, stroke; ^c^ PHQ4: Patient Health Questionnaire for Depression and Anxiety.

**Table S5: Clinical Outcomes Pre- and Post-Patient Watch**

|  | Pre-Patient Watch | | | | Post-Patient Watch | | | |
| --- | --- | --- | --- | --- | --- | --- | --- | --- |
|  | Triage Category (resuscitation/emergency/ urgent) (n = 10) | | Triage Category (semi-urgent/non-urgent) (n = 27) | | Triage Category (resuscitation/emergency/ urgent) (n = 4) | | Triage Category (semi-urgent/non-urgent) (n = 33) | |
| *Emergency department data* | Baseline | Follow-up | Baseline | Follow-up | Baseline | Follow-up | Baseline | Follow-up |
| ED presentations, mean (SD) | 4.1 (3.0) | 1.6 (3.1) | 1.4 (0.8) | 0.8 (1.3) | 5.8 (4.5) | 3.3 (4.6) | 1.7 (1.0) | 0.7 (1.2) |
| Triage category, n (%) | n = 41*^a^* | n = 16*^a^* | n = 36*^a^* | n = 20*^a^* | n = 2 4*^a^* | n = 15*^a^* | n = 53*^a^* | n = 21*^a^* |
| Resuscitation | 0 | 0 | 0 | 1 (5) | 0 | 1 (7) | 0 | 1 (5) |
| Emergency | 3 (7) | 1 (6) | 18 (50) | 9 (45) | 3 (13) | 8 (53) | 18 (34) | 9 (43) |
| Urgent | 19 (46) | 10 (63) | 18 (50) | 9 (45) | 12 (50) | 5 (33) | 25 (47) | 11 (52) |
| Semi-urgent | 18 (44) | 4 (25) | 0 | 1 (5) | 8 (33) | 1 (7) | 10 (19) | 0 |
| Non-urgent | 1 (2) | 1 (6) | 0 | 0 | 1 (4) | 1 (7) | 0 | 0 |
| Number of admissions, mean (SD) | 2.0 (1.1) | 0.5 (1.0) | 1.2 (0.9) | 0.5 (0.9) | 2.8 (1.0) | 0.8 (1.5) | 1.3 (0.9) | 0.5 (0.9) |
| *Self-reported data* |  |  |  |  |  |  |  |  |
| Number of GP^b^ visits, mean (SD) | 11.4 (5.6) | 10.0 (11.0) | 6.8 (5.1) | 5.6 (3.8) | 9.5 (4.2) | 15.3 (16.9) | 7.9 (5.7) | 5.8 (3.6) |
| Number of specialist clinician visits, mean (SD) | 4.7 (6.5) | 2.5 (2.1) | 3.2 (3.7) | 1.9 (2.5) | 7.0 (8.8) | 4.3 (1.7) | 3.2 (3.8) | 1.8 (2.4) |
| AQol-8D^c^, mean (SD) |  |  |  |  |  |  |  |  |
| Independent living domain | 0.50 (0.13) | 0.52 (0.11) | 0.74 (0.16) | 0.69 (0.18) | 0.53 (0.12) | 0.58 (0.15) | 0.69 (0.53) | 0.65 (0.18) |
| Happiness dimension | 0.66 (0.18) | 0.71 (0.15) | 0.73 (0.14) | 0.71 (0.15) | 0.72 (0.07) | 0.78 (0.13) | 0.71 (0.16) | 0.70 (0.15) |
| Mental health dimension | 0.56 (0.12) | 0.59 (0.13) | 0.62 (0.12) | 0.61 (0.13) | 0.55 (0.07) | 0.58 (0.05) | 0.61 (0.13) | 0.61 (0.13) |
| Coping dimension | 0.66 (0.17) | 0.67 (0.17) | 0.72 (0.16) | 0.73 (0.15) | 0.68 (0.14) | 0.69 (0.18) | 0.70 (0.16) | 0.71 (0.15) |
| Relationships dimension | 0.59 (0.12) | 0.58 (0.08) | 0.67 (0.15) | 0.67 (0.16) | 0.60 (0.09) | 0.63 (0.09) | 0.65 (0.15) | 0.65 (0.15) |
| Self-worth dimension | 0.63 (0.19) | 0.64 (0.18) | 0.72 (0.17) | 0.75 (0.12) | 0.68 (0.07) | 0.64 (0.11) | 0.69 (0.19) | 0.73 (0.15) |
| Pain dimension | 0.40 (0.27) | 0.34 (0.22) | 0.62 (0.25) | 0.62 (0.27) | 0.31 (0.14) | 0.33 (0.20) | 0.60 (0.27) | 0.57 (0.28) |
| Senses dimension | 0.83 (0.14) | 0.84 (0.10) | 0.86 (0.12) | 0.85 (0.11) | 0.86 (0.04) | 0.91 (0.08) | 0.85 (0.13) | 0.84 (0.10) |
| Super-dimension: mental | 0.24 (0.15) | 0.25 (0.12) | 0.33 (0.17) | 0.32 (0.16) | 0.24 (0.06) | 0.26 (0.08) | 0.31 (0.18) | 0.31 (0.16) |
| Super-dimension: physical | 0.35 (0.14) | 0.33 (0.14) | 0.56 (0.19) | 0.54 (0.21) | 0.32 (0.10) | 0.36 (0.15) | 0.52 (0.20) | 0.50 (0.22) |
| Utility score | 0.45 (0.18) | 0.46 (0.16) | 0.61 (0.20) | 0.61 (0.19) | 0.46 (0.09) | 0.49 (0.14) | 0.58 (0.21) | 0.58 (0.20) |
| PACIC^d^, mean (SD) |  |  |  |  |  |  |  |  |
| Patient activation | 2.7 (1.4) | 3.3 (1.4) | 3.4 (1.3) | 3.1 (1.6) | 3.1 (1.5) | 3.6 (1.3) | 3.2 (1.4) | 3.1 (1.5) |
| Delivery system design/decision support | 3.2 (1.6) | 3.0 (1.1) | 4.0 (1.0) | 3.6 (1.3) | 4.2 (1.1) | 3.9 (1.1) | 3.7 (1.2) | 3.4 (1.3) |
| Goal setting/tailoring | 3.2 (1.4) | 3.3 (1.1) | 3.5 (1.3) | 3.3 (1.5) | 3.8 (1.2) | 3.8 (1.0) | 3.4 (1.4) | 3.2 (1.4) |
| Problem-solving/contextual counselling | 2.8 (1.3) | 2.9 (1.3) | 3.3 (1.3) | 3.5 (1.5) | 3.8 (1.2) | 3.8 (1.0) | 3.1 (1.3) | 3.2 (1.5) |
| Follow-up/coordination | 2.8 (1.2) | 2.5 (1.3) | 3.2 (1.2) | 2.9 (1.3) | 3.8 (1.1) | 3.3 (1.4) | 3.0 (1.2) | 2.8 (1.3) |
| Total | 2.9 (1.2) | 3.0 (1.0) | 3.4 (1.1) | 3.3 (1.3) | 3.7 (1.1) | 3.7 (1.0) | 3.2 (1.1) | 3.1 (1.2) |

^a^ Total emergency department presentations; ^b^ GP: General Practitioner; ^c^ AQoL-8D: Assessment of Quality of Life-8D; ^d^ PACIC: Patient Assessment of Chronic Illness Care.

**Table S6: Patient Watch Program Data**

|  | Baseline | | Follow-up | |
| --- | --- | --- | --- | --- |
|  | Triage Category (resuscitation/ emergency/ urgent) (n = 10) | Triage Category (semi-urgent/ non-urgent) (n = 27) | Triage Category (resuscitation/ emergency/ urgent) (n = 4) | Triage Category (semi-urgent/ non-urgent) (n = 33) |
| Mode of entry, n (%) |  |  |  |  |
| Referral | 7 (70) | 17 (63) | 1 (25) | 10 (30) |
| HLCC Extract (algorithm) | 3 (30) | 10 (37) | 3 (75) | 23 (70) |
| TNS^a^ worker, mean (SD) |  |  |  |  |
| Number of calls | 18.3 (8.2) | 14.5 (4.4) | 14.8 (2.8) | 15.6 (6.1) |
| Duration per call (minutes) | 17.0 (7.5) | 12.3 (3.7) | 12.8 (4.4) | 13.7 (5.5) |
| Did not answer | 4.1 (3.3) | 3.9 (3.7) | 3.5 (2.4) | 4.0 (3.6) |
| Health coach, mean (SD) |  |  |  |  |
| Number of calls | 8.5 (3.3) | 7.1 (3.7) | 7.0 (2.2) | 7.5 (3.8) |
| Duration per call (minutes) | 57.7 (15.4) | 59.0 (24.4) | 57.2 (15.4) | 58.8 (23.0) |
| Did not answer | 0.8 (1.2) | 1.4 (2.6) | 1.3 (1.9) | 1.2 (2.4) |
| Problems and actions, mean (SD) | 2.1 (2.6) | 1.8 (2.4) | 0.75 (1.0) | 2.0 (2.5) |
| Status at follow-up, n (%) |  |  |  |  |
| Continuing | 9 (90) | 19 (70) | 4 (100) | 24 (73) |
| Discharge | 1 (10) | 8 (30) | 0 | 9 (27) |

^a^ Tele-navigator support.

**Mode of entry into Patient Watch**

**Table S7: Characteristics of Participants**

|  | Referral, n = 24 | HLCC algorithm, n = 13 |
| --- | --- | --- |
| Age, mean (SD) | 72.0 (12.8) | 69.9 (10.1) |
| Male, n (%) | 11 (46) | 9 (69) |
| Education level, n (%) |  |  |
| Primary school | 2 (8) | 1 (8) |
| Secondary school | 9 (38) | 3 (23) |
| Further education (university/vocational) | 13 (54) | 9 (69) |
| Living arrangement, n (%) |  |  |
| Live alone | 12 (50) | 2 (15) |
| Partner/spouse | 5 (21) | 9 (69) |
| Other (e.g., siblings, share house) | 7 (29) | 2 (15) |
| Australian healthcare concession card, n (%) |  |  |
| No concession card | 3 (13) | 3 (23) |
| Concession card | 21 (88) | 10 (77) |
| Main health condition for program participation, n (%) |  |  |
| COPD^a^ | 8 (33) | 2 (15) |
| Heart failure, chronic | 5 (21) | 3 (23) |
| Hypertension | 2 (8) | 3 (23) |
| Diabetes Type 2 | 0 | 2 (15) |
| Osteoarthritis | 2 (8) | 0 |
| Other^b^ | 7 (29) | 3 (23) |
| Mental health diagnosis, n (%) | 11 (46) | 4 (31) |
| Number of chronic conditions, mean (SD) | 6.7 (2.7) | 5.2 (2.6) |
| PHQ4^c^, mean (SD) | 2.8 (3.3) | 4.0 (3.3) |

^a^ COPD: Chronic Obstructive Pulmonary Disease; ^b^ Other: Bronchiectasis, lymphoedema, acquired brain injury, pneumonia, schizophrenia, chronic pain, asthma, urinary incontinence, stroke; ^c^ PHQ4: Patient Health Questionnaire for Depression and Anxiety.

**Table S8: Clinical Outcomes Pre- and Post-Patient Watch**

|  | Referral, n = 24 | | HLCC algorithm, n = 13 | |
| --- | --- | --- | --- | --- |
|  | Pre-Patient Watch | Post-Patient Watch | Pre-Patient Watch | Post-Patient Watch |
| *Emergency department data* | *n = 39 ^a^* | *n = 18 ^a^* | *n = 38 ^a^* | *n = 18 ^a^* |
| ED presentations, mean (SD) | 1.8 (1.4) | 0.8 (1.1) | 2.9 (2.8) | 1.4 (2.8) |
| Triage category, n (%) |  |  |  |  |
| Resuscitation | 0 | 1 (6) | 0 | 0 |
| Emergency | 9 (23) | 4 (22) | 12 (32) | 6 (33) |
| Urgent | 20 (51) | 11 (61) | 17 (45) | 8 (44) |
| Semi-urgent | 10 (26) | 2 (11) | 8 (21) | 3 (17) |
| Non-urgent | 0 | 0 | 1 (3) | 1 (6) |
| Number of admissions, mean (SD) | 1.0 (0.8) | 0.4 (0.7) | 2.2 (2.8) | 0.6 (1.2) |
| *Self-reported data* |  |  |  |  |
| Number of GP^b^ visits, mean (SD) | 7.5 (5.1) | 6.1 (3.4) | 9.1 (6.4) | 8.1 (10.4) |
| Number of specialist clinician visits, mean (SD) | 4.3 (5.5) | 2.1 (2.6) | 2.3 (1.7) | 2.0 (2.1) |
| AQol-8D^c^, mean (SD) |  |  |  |  |
| Independent living domain | 0.64 (0.19) | 0.60 (0.17) | 0.73 (0.18) | 0.73 (0.17) |
| Happiness dimension | 0.70 (0.17) | 0.69 (0.16) | 0.73 (0.12) | 0.74 (0.12) |
| Mental health dimension | 0.61 (0.13) | 0.58 (0.13) | 0.59 (0.11) | 0.65 (0.12) |
| Coping dimension | 0.70 (0.17) | 0.70 (0.16) | 0.71 (0.15) | 0.75 (0.13) |
| Relationships dimension | 0.65 (0.13) | 0.64 (0.15) | 0.65 (0.17) | 0.66 (0.15) |
| Self-worth dimension | 0.70 (0.20) | 0.71 (0.14) | 0.67 (0.15) | 0.76 (0.16) |
| Pain dimension | 0.54 (0.27) | 0.51 (0.27) | 0.62 (0.27) | 0.60 (0.30) |
| Senses dimension | 0.86 (0.13) | 0.85 (0.11) | 0.85 (0.10) | 0.86 (0.10) |
| Super-dimension: mental | 0.31 (0.17) | 0.29 (0.15) | 0.29 (0.17) | 0.34 (0.15) |
| Super-dimension: physical | 0.48 (0.21) | 0.44 (0.19) | 0.54 (0.20) | 0.55 (0.24) |
| Utility score | 0.57 (0.22) | 0.54 (0.20) | 0.57 (0.18) | 0.63 (0.17) |
| PACIC^d^, mean (SD) |  |  |  |  |
| Patient activation | 3.2 (1.4) | 3.4 (1.4) | 3.2 (1.4) | 2.7 (1.6) |
| Delivery system design/decision support | 3.7 (1.2) | 3.5 (1.3) | 3.9 (1.2) | 3.4 (1.3) |
| Goal setting/tailoring | 3.4 (1.3) | 3.3 (1.3) | 3.4 (1.5) | 3.3 (1.5) |
| Problem-solving/contextual counselling | 3.2 (1.3) | 3.2 (1.5) | 3.2 (1.5) | 3.5 (1.4) |
| Follow-up/coordination | 3.0 (1.2) | 2.9 (1.2) | 3.2 (1.1) | 2.7 (1.3) |
| Total | 3.3 (1.1) | 3.2 (1.2) | 3.3 (1.1) | 3.1 (1.3) |

^a^ Total emergency department presentations; ^b^ GP: General Practitioner; ^c^ AQoL-8D: Assessment of Quality of Life-8D; ^d^ PACIC: Patient Assessment of Chronic Illness Care.

**Table S9: Patient Watch Program Data**

|  | Referral n = 24 | HLCC algorithm n = 13 |
| --- | --- | --- |
| TNS*^a^* worker, mean (SD) |  |  |
| Number of calls | 16.5 (6.4) | 13.8 (4.1) |
| Duration per call (minutes) | 14.2 (5.9) | 12.4 (4.2) |
| Did not answer | 4.0 (3.4) | 3.8 (3.9) |
| Health coach, mean (SD) |  |  |
| Number of calls | 8.2 (3.9) | 6.1 (2.8) |
| Duration per call (minutes) | 57.6 (19.4) | 60.5 (27.2) |
| Did not answer | 1.3 (2.7) | 1.1 (1.5) |
| Problems and actions, mean (SD) | 2.2 (2.7) | 1.3 (1.7) |
| Status at follow-up, n (%) |  |  |
| Continuing | 20 (83) | 8 (62) |
| Discharge | 4 (17) | 5 (39) |

*^a^* Tele-navigator support.
